# Prevalence, Severity, Concomitant Factors, and Natural Trajectory of Insomnia in Patients with Long COVID

**DOI:** 10.3390/jcm14176114

**Published:** 2025-08-29

**Authors:** Jamie Hansel Robinson, Halle Bakir, Alicia Shanti James, Marquita S. Brooks, Stephen J. Thomas, Kristine L. Lokken

**Affiliations:** 1 Department of Psychiatry and Behavioral Neurobiology, The University of Alabama at Birmingham, Birmingham, AL 35233, USA; 2 Department of Graduate Psychology, Pacific University, Forest Grove, OR 97116, USA; 3 Department of Psychology, The University of Alabama at Birmingham, Birmingham, AL 35233, USA

**Keywords:** COVID-19, Long COVID insomnia, Long COVID, mental health

## Abstract

**Background/Objective:** Insomnia is a clinically important symptom in Long COVID; however, few studies have addressed the presentation and course of insomnia symptoms in patients with Long COVID. **Methods:** The Insomnia Severity Index (ISI) was administered as part of a comprehensive baseline neuropsychological evaluation (Time 1) for patients with Long COVID at an Academic Medical Center (AMC). Data were gathered on 172 consecutively referred patients between the dates of November 2020 and May 2022. The mean age of patients at Time 1 was 49 years (range: 18 to 78), with a mean of 15 years of education. Patients were 70% female and 30% male and identified as White/Caucasian (78%), Black/African American (21%), or American Indian (1%). Patients’ severity of COVID-19 infection and self-reported emotional, somatic, cognitive, and fatigue symptoms were also gathered to identify concomitant risk factors for insomnia in Long COVID. Patients were then followed to observe the natural trajectory of insomnia complaints in Long COVID, with the Time 2 evaluation a mean of 9 months after the Time 1 evaluation. **Results:** Seventy-eight percent of Long COVID patients reported insomnia symptoms at Time 1, with 30% reporting Subthreshold Insomnia symptoms (ISI Score = 8–14), 30% reporting Moderate Insomnia symptoms (ISI Score = 15–21), and 18% reporting Severe Clinical Insomnia (ISI Score = 22–28). Severity of acute COVID-19 infection was not correlated with severity of insomnia in Long COVID; however, being non-white (*r* = 0.24, *n* = 172, *p* < 0.01) and having higher self-reported levels of anxiety (*r* = 0.41, *n* = 172, *p* < 0.01), depression (*r* = 0.52, *n* = 172, *p* < 0.01), perceived stress (*r* = 0.38, *n* = 172, *p* < 0.01), somatic symptoms (*r* = 0.51, *n* = 172, *p* < 0.01), cognitive failures, and fatigue were significantly correlated with insomnia symptoms. Insomnia was also significantly correlated with lower global cognitive function (*r* = 0.51, *n* = 172, *p* < 0.01) and lower cognitive flexibility (*r* = −0.17, *n* = 172, *p* < 0.05). There was a statistically significant decrease in reported ISI scores from Time 1 to Time 2 (*t* = −3.04; *p* = 0.003); however, ISI mean scores at both Time 1 (ISI Score = 14) and Time 2 (ISI Score = 12) remained in the Subthreshold Insomnia range (ISI score 8–14). **Conclusions:** Findings suggest that a large majority of Long COVID patients experience insomnia symptoms. Additionally, insomnia symptoms did not dissipate over time in a clinically meaningful way and were highly correlated with reduced global cognitive function, reduced cognitive flexibility, and higher levels of reported mood symptoms, fatigue, somatic symptoms, and experience of cognitive failures. Thus, there is a pressing need for intervention strategies to treat insomnia in Long COVID patients.

## 1. Introduction

### 1.1. Long COVID

As of this writing, there have been 777,726,897 cases of COVID-19 worldwide and 103,436,829 cases in the United States [1]. Data suggests approximately 10% of COVID-19 cases develop Long COVID [2]. Long COVID refers to a myriad of ongoing, relapsing, or new symptoms across multiple organ systems that present or persist for more than 8 weeks and arise within 12 weeks of an acute COVID-19 infection [3,4]. The symptoms are numerous and varied, with neuropsychiatric complications such as fatigue, insomnia, brain fog, and cognitive issues being among the most impactful and likely to persist [5].

### 1.2. Insomnia in the Context of Long COVID

Insomnia affects about 10–30% of the general population. It is associated with a plethora of adverse mental health complications, including increased risk of depression, anxiety disorders, suicide, substance use disorders, and objective and subjective cognitive impairment [6,7,8,9]. Globally, the COVID-19 pandemic resulted in increased rates of the cumulative prevalence of subthreshold insomnia, along with increased population rates of anxiety, depression, post-traumatic stress disorder (PTSD), and stress. In contrast, the prevalence of moderate and severe insomnia remained stable [10]. Independent of the effects of the pandemic, insomnia is a symptom of acute COVID-19 infection and is associated with longer recovery times to return to pre-infection daily functioning [11]. Insomnia is also a central component of Long COVID, yet limited research describes the presentation of insomnia in those with Long COVID.

A study by Chen et al. [12] found a bidirectional relationship between insomnia and Long COVID in that COVID-19 patients with pre-pandemic insomnia had a greater risk of developing Long COVID compared to those without a history of insomnia (71% versus 51%), and patients without a history of pre-pandemic insomnia who developed Long COVID were at increased risk of developing insomnia symptoms (61%) as a part of their clinical presentation. Mood symptoms, sleep disturbance, and fatigue are common in Long COVID and also salient predictors of decreased cognitive test performance [13]. Given the associations between Long COVID, mental health issues, cognition, and insomnia, further study is necessary to better understand the prevalence, severity, concomitant factors, and natural trajectory of insomnia in Long COVID. Examination of such issues can help inform clinical efforts focused on the prevention, early intervention, and management of insomnia in Long COVID.

### 1.3. Infection Severity and Long COVID Symptoms

While the severity of acute infection has been thought to influence the prevalence of certain Long COVID symptoms, the literature is equivocal on the relationship between these factors. For example, Merikanto et al. [14] found that individuals with moderate or severe COVID-19 infections experienced the highest prevalence of long-lasting symptoms, including prolonged sleep issues (i.e., daytime sleepiness, insomnia, and fatigue), cognitive deficits, and concentration difficulties. However, Premraj et al. [15] reported that patients who were hospitalized with acute COVID-19 infection (i.e., “severe” infection by 2020 WHO guidelines [16]) were less likely to develop neurological issues, mood disturbance, fatigue, and sleep disturbance in the initial months after infection than those who were not hospitalized. Alternatively, a systematic review by Marchi et al. [17] found no significant relationship between COVID-19 infection severity and subsequent psychiatric symptoms, including depression, anxiety, and sleep disturbances. More recently, Kadl et al. [18] found no association between persistent insomnia and initial COVID-19 infection severity. Taken together, this literature highlights a need for further research examining the association between acute COVID-19 infection severity and Long COVID symptoms, particularly insomnia.

### 1.4. Risk Factors for Insomnia in Long COVID

Certain factors may predispose an individual to experience increased severity of Long COVID symptoms, including increased severity of insomnia. Evidence suggests a role for demographic and mental health variables, with Kadl et al. [18] reporting that lower age, female sex, being non-white and of non-Hispanic ethnicity, as well as higher levels of reported anxiety and depression, were all significantly associated with higher levels of reported insomnia in patients following COVID-19 infection. Except for lower age, these factors are consistent with the risks of chronic insomnia in the general population [7]. Other research indicates that those who had worse scores on the Insomnia Severity Index (ISI) and Pittsburgh Sleep Quality Index (PSQI) and shorter sleep duration before COVID-19 infection were at higher risk for developing Long COVID symptoms and generally experienced a longer recovery period after acute infection [11]. Identifying those most at risk for developing insomnia post-COVID infection can help to create targeted prevention and early intervention strategies to thwart the onset of or persistence of insomnia symptoms following COVID-19 infection.

### 1.5. Natural Trajectory of Insomnia in Long COVID

Few studies adequately report on the longitudinal course of insomnia in patients with Long COVID. Gong et al. [19] followed insomnia symptoms of older adults during the first year of the COVID-19 pandemic, finding that >1 in 3 experienced persistent subthreshold or clinically significant insomnia; however, this study did not specify whether participants experienced COVID-19 infection or Long COVID. Kadl et al. [18] found that the prevalence of clinical insomnia decreased from 50% to 42% over time after COVID-19 infection, with reported anxiety and PTSD strongly correlating with persistent insomnia; however, the analyses included typical COVID cases in addition to Long COVID cases. To our knowledge, there has yet to be a study solely examining prevalence rates of insomnia in Long COVID patients, further emphasizing the vital need for more research.

Interestingly, research suggests that the polysomnography of Long COVID insomnia is similar to that of chronic insomnia in its detrimental impact on sleep efficiency, duration, and waking after sleep onset [20]. These findings suggest that efficacious treatments for chronic insomnia may be beneficial in treating those with insomnia related to Long COVID. Specifically, Cognitive Behavioral Therapy for Insomnia (CBT-I) has been established as an effective and safe intervention for a wide variety of populations, including those with comorbid psychiatric conditions [21,22], and may ultimately be a promising intervention for insomnia secondary to Long COVID.

### 1.6. Current Study

Overall, the Long COVID landscape is marked by enduring sleep-related challenges, the symptoms of which may manifest differently based on various demographic and infection-related factors. Given the high prevalence of insomnia and its widespread impact across physical, mental, and cognitive disorders, there is a critical need for research to examine insomnia in the context of Long COVID, especially in light of the paucity of available research. This study is unique in that it employs a robust longitudinal, multidomain neuropsychological approach to examining insomnia-specific symptoms in Long COVID. Specifically, the current study aimed to (1) determine the prevalence and severity of insomnia in patients with Long COVID; (2) examine the relationship between the severity of acute COVID-19 infection and insomnia severity in Long COVID; (3) determine the concomitant risk factors (e.g., patient demographics, anxiety, depression, stress, somatic symptoms, fatigue, and perceived and objective cognitive function) that are related to insomnia in patients with Long COVID; and (4) examine the natural trajectory of insomnia symptoms in Long COVID.

Institutional Review Board (IRB) approval was obtained through the University of Alabama at Birmingham (UAB) Institutional Review Board for Human Use (IRB), as protocol IRB-300007334: Post-COVID Neurocognitive Functioning Outpatient Registry. Data were gathered for clinical care and examined retrospectively. Given this, it was an expedited approval for a non-clinical trial. Approval was granted on 17 December 2021 and extended through 20 March 2028.

## 2. Materials and Methods

### 2.1. Patients

Patients in a large Academic Medical Center (AMC) were referred for neuropsychological assessment due to persistent mood and cognitive symptoms more than 90 days post-COVID-19 infection. Patients were either self-referred, referred via the university’s multi-disciplinary Post-COVID Treatment Program, or referred by primary care physicians or other specialty providers. One hundred and seventy-two consecutively referred patients completed the baseline neuropsychological evaluation between November 2020 and May 2022. Patients were included in the dataset if they met criteria for having Long COVID. All patients referred were deemed to meet criteria, and no patients were excluded. All patients underwent a standard of care clinical evaluation, consisting of a clinical diagnostic interview, completion of subjective self-report questionnaires (including the ISI), and a full battery of neuropsychological testing administered by a trained psychometrist via telehealth methods. At the time of this writing, eighty-nine patients (52%) returned to the clinic to repeat testing (Time 2) and were included in the Time 2 analyses, with data collection ongoing. Time 2 evaluations were conducted a mean of 9 months after the Time 1 evaluations. Institutional Review Board (IRB) approval was obtained to examine the data retrospectively. Patient demographics are presented in Table 1. Of the 172 patients who completed the baseline (Time 1) evaluation, on average, patients were 49 years of age (range: 18 to 78), primarily female (70%), and of White/Caucasian (78%), Black/African American (21%), or American Indian (1%) race, with a mean of 15 years of education. For the 89 patients who returned for follow-up (Time 2) evaluation, on average, patients were 51 years of age, primarily female (69%), and of White/Caucasian (77%) or Black/African American (22%) race, with a mean of 16 years of education. Patient demographics were similar at Time 1 and Time 2.

### 2.2. Measures

For this study, insomnia was the primary outcome variable as measured by the ISI.

### 2.3. Comprehensive Questionnaire Battery

Data for the questionnaire battery was gathered using the online survey provider Research Electronic Data Capture (REDCap) along with additional demographic, medical history, COVID-19 infection details, and other pertinent patient data. All questionnaires and cognitive measures were well validated and reliable, and administration was consistent with state-of-the-art psychological and neuropsychological assessment techniques.

Sleep

The ISI was used to measure sleep quality and insomnia symptoms. The ISI is composed of seven items in which patients rank the severity of the insomnia symptoms in the last two weeks. The first three items are rated on a 5-point Likert scale to describe the severity (0 = *None*, 1 = *Mild*, 2 = *Moderate*, 3 = *Severe*, 4 = *Very Severe*) of difficulty falling asleep, staying asleep, and problems with waking up too early. The four remaining items are also rated on a 5-point Likert scale and ask a patient to report how satisfied they are with their sleep pattern (*Very Satisfied* to *Very Dissatisfied*), how noticeably their sleep problems are impacting their quality of life (*Not at all Noticeable* to *Very Much Noticeable*), how worried or distressed they are about their sleep pattern (*Not at all Worried* to *Very Much Worried*), and to what extent they believe their sleep pattern interferes with their daily functioning (*Not at all Interfering* to *Very Much Interfering*). Total scores of 0 to 7 are considered within normal limits, scores of 8 to 14 are categorized as subthreshold insomnia, scores of 15 to 21 are categorized as clinical insomnia with moderate severity, and scores of 22 to 28 are categorized as Severe Clinical Insomnia [23,24].

Anxiety

The Generalized Anxiety Disorder—7 item (GAD-7) [25] was used to measure anxiety symptoms experienced in the last two weeks. Seven items measure anxiety symptoms, and one item measures the impact the symptoms endorsed above have on a person’s work and family obligations. The first seven questions are scored on a 4-point scale of how often the patient was bothered by each symptom (0 = *Not at all*, 1 = *Several days*, 2 = *Over half the days*, 3 = *Nearly every day*). The final question measures difficulty on a 4-point scale (0 = *Not difficult at all*, 1 = *Somewhat difficult*, 2 = *Very difficult*, 3 = *Extremely difficult*). A score of 0 through 4 is considered within normal limits, scores between 5 and 9 are suggestive of mild anxiety, scores between 10 and 14 are considered indicative of moderate anxiety, and scores of 15 and higher suggest severe anxiety.

Depression

The Patient Health Questionnaire—8 item (PHQ-8) [26] was used to measure depression symptoms experienced in the last two weeks. Eight items measure symptoms of depression, and a final item asks the patient to rate the impact of the depression symptoms on performing in their job and family obligations. The eight questions are scored on a 4-point scale of how often they had been bothered in the past two weeks (0 = *Not at all*, 1 = *Several days*, 2 = *More than half the days*, 3 = *Nearly every day*). Scores from 0 to 4 suggest minimal depressive symptoms, scores of 5 to 9 suggest mild depressive symptoms, scores of 10 to 14 suggest moderate depressive symptoms, scores of 15 to 19 suggest moderately severe depressive symptoms, and scores of ≥20 suggest severe depressive symptoms.

Stress

The Perceived Stress Scale (PSS) [27] was used to measure a patient’s experience of stress at the time of evaluation. There are ten stress symptom items on the questionnaire, and patients are asked to rate their experience of the symptoms in the past month. Items are scored on a 5-point Likert scale from *never* to *very often*. Scores from 0 to 13 are considered low perceived stress, scores of 14 to 26 are considered moderate perceived stress, and scores of 27 to 40 are considered indicative of high perceived stress.

Somatic Symptoms

The Patient Health Questionnaire—15 item (PHQ-15) [28,29] was used to measure somatic symptoms. A total of 15 items describing specific physical symptoms are scored on a 3-point Likert scale from *Not bothered at all* to *Bothered a lot*. Scores between 0 and 4 are considered within normal limits, scores between 5 and 9 are considered low somatic symptom severity, scores between 10 and 14 are considered medium somatic symptom severity, and scores of 15 and above are considered high somatic symptom severity.

Perceived Cognitive Function

The Cognitive Failures Questionnaire (CFQ) [30,31] was used to measure a patient’s perceived cognitive functioning ability. CFQ is composed of 24 cognitive error items (e.g., Do you forget why you went from one part of the house to the other? Do you find you forget what you came to the shops to buy?) to which the patient responds how often each error has been made within the previous six months. All items are scored on a 5-point Likert scale from *Never* to *Very Often*. Scores of 32 and higher are considered indicative of perceived cognitive impairment.

Fatigue

The Chalder Fatigue Scale (CFS) [32] was used to measure a patient’s fatigue symptoms in recent weeks. The CFS is composed of 11 total items. The first ten items require the patient to describe any problems with feeling tired, weak, or lacking energy in the past week. The CFS specifically asks the subject to compare their current fatigue to the energy levels when they last felt well. Items were scored on a 4-point Likert scale (0 = *Less than usual*, 1 = *No more than usual*, 2 = *More than usual*, 3 = *Much more than usual*). The final item asks patients about their memory and is also scored on a 4-point Likert scale (0 = *Better than usual*, 1 = *No worse than usual*, 2 = *Worse than usual*, 3 = *Much worse than usual*). Total scores are then compared to the mean and standard deviation of the original study’s community sample to create a z-score.

### 2.4. Comprehensive Cognitive Battery

Patients completed a full neuropsychological battery via video telehealth. Cognitive testing was completed by trained psychometricians under the supervision of a licensed clinical neuropsychologist. Cognitive assessment included testing of major cognitive domains, including estimated premorbid function, global cognitive function, attention, concentration, and working memory; processing speed; executive functions; language ability; visuospatial construction; and verbal and nonverbal learning and memory, in addition to embedded measures of effort. A full list of cognitive measures is provided in Table 2.

### 2.5. Statistical Analyses

Frequency descriptive analyses were performed to report prevalence and severity of insomnia at Time 1. Additionally, frequency distributions were provided for acute Long COVID infection severity. We assessed correlational relationships between insomnia symptoms and infection severity, number of infections, and cognitive performance. Correlation analyses were conducted to examine relationships among ISI, demographic variables, neurocognitive measures, and self-reported mood. Lastly, repeated measures statistical analyses were performed to assess mean differences between Time 1 and Time 2 insomnia, emotional, and cognitive measures.

## 3. Results

### 3.1. Prevalence and Severity of Insomnia at Time 1 Evaluation

Complete baseline (Time 1) data from 6 patients were missing for ISI, for a total sample of 166 included in analyses at Time 1. Time 1 ISI analyses revealed that 33 of 166 patients (19%) were categorized as experiencing No Insomnia (ISI score 0–7). A majority of patients reported mild to moderate insomnia symptoms, with 51 patients (30%) falling into the Subthreshold Insomnia category (ISI score 8–14) and 51 patients (30%) meeting the clinical threshold of Moderate Insomnia (ISI score 15–21). The remaining 31 patients (18%) reported symptoms consistent with Severe Clinical Insomnia (ISI score 22–28). Taken together, 78% of Long COVID patients endorsed insomnia symptoms, with nearly 50% of Long COVID patients reporting significant insomnia (ISI ≥ 15) at baseline evaluation—see Table 1.

### 3.2. Severity of Acute COVID-19 Infection and Insomnia Severity

Acute COVID-19 Infection Severity Rating was recorded for 164 of the 172 patients included in this database, with categories for asymptomatic (defined as no initial symptoms), mild (defined as symptomatic, but no difficulty breathing), moderate (defined as difficulty breathing), severe (defined as hospitalized and/or requiring oxygen), and critical (defined as hospitalized in the ICU), based on WHO guidance for COVID-19 infection severity [16]. See Table 3 for the frequency of infection severity ratings for this sample.

The infection severity of acute, initial COVID-19 infection was not correlated with the severity of insomnia on the ISI. Specifically, there was no significant relationship between infection severity rating and ISI scores at Time 1, *r* = 0.08, *n* = 162, *p* = 0.29. Additionally, infection severity exhibited no significant relationship with ISI scores at Time 2, *r* = 0.12, *n* = 84, *p* = 0.28. However, there was a significant strong positive correlation between Time 1 and Time 2 ISI scores, *r* = 0.64, *n* = 84, *p* < 0.001.

The number of COVID-19 infections was also recorded for 103 of the 172 patients at baseline (Time 1) evaluation. For those with a known number of COVID-19 infections, the majority of patients had only one COVID-19 infection (40%), followed by those with two COVID-19 infections (13%) and those with ≥3 known COVID-19 infections (7%). There was no significant relationship between the number of reported COVID-19 infections and insomnia severity at Time 1, *r* = 0.03, *n* = 102, *p* = 0.78, or Time 2, *r* = −0.01, *n* = 55, *p* = 0.97.

Further, there was no significant relationship between acute COVID-19 infection severity rating and global cognitive performance, as measured by the Montreal Cognitive Assessment (MoCA) [33], at either Time 1, *r* = −0.03, *n* = 164, *p* = 0.72, or Time 2, *r* = −0.14, *n* = 87, *p* = 0.21, and no significant relationship between number of reported COVID-19 infections and global cognitive performance, as measured by MoCA at Time 1, *r* = −0.00, *n* = 103, *p* = 1.0, or Time 2, *r* = 0.17, *n* = 57, *p* = 0.21. However, global cognitive performance, as measured by the MoCA, was significantly correlated with ISI at both Time 1, *r* = −0.21, *n* = 166, *p* < 0.01, and Time 2, *r* = −0.32, *n* = 85, *p* < 0.003.

### 3.3. Concomitant Factors

Correlation analyses were conducted to examine relationships among ISI scores and other variables (Table 4). At baseline (Time 1), non-white race (*r* = 0.24, *n* = 172, *p* < 0.01), lower global cognition on the MoCA (*r* = −0.21, *n* = 172, *p* < 0.01), poorer performance on the DKEFS Inhibition/Switching task (*r* = −0.17, *n* = 172, *p* < 0.05), higher levels of anxiety on the GAD-7 (*r* = 0.41, *n* = 172, *p* < 0.01), higher levels of depression on the PHQ-8 (*r* = 0.52, *n* = 172, *p* < 0.01), higher levels of perceived stress on the PSS (*r* = 0.38, *n* = 172, *p* < 0.01), and higher levels of somatic symptoms on the PHQ-15 (*r* = 0.51, *n* = 172, *p* < 0.01) were significantly associated with higher ISI scores. Gender, age, years of education, Oral Symbol Digit Modalities Test, WAIS-IV Digit Span, Oral Trail Making Test Part B, DKEFS Inhibition, CVLT-3 Brief Form Trials 1–4, CVLT-3 Brief Form Long Delay Free Recall, RBANS Story Memory Immediate, and RBANS Story Memory Delay scores did not significantly correlate with ISI scores.

### 3.4. Insomnia Trajectory

Eighty-nine patients returned for the Time 2 evaluation. Four patients did not complete the ISI at Time 2, so a total sample of 85 patients was included in Time 2 ISI analyses. At Time 2, 24 patients (27%) reported no insomnia symptoms (ISI score 0–7), whereas the remaining 61 patients (72%) reported some insomnia symptoms, with 27 (30%) meeting the guideline thresholds for Subthreshold Insomnia (ISI score 8–14), 23 (26%) reporting Moderate Insomnia (ISI score 15–21), and 11 (12%) reporting symptoms consistent with Severe Clinical Insomnia (ISI score 22–28)—see Table 1.

The mean time between the Time 1 evaluation and the Time 2 evaluation was 9 months and ranged from 5 to 21 months. The ISI average score (SD) was 14 (6.48) at baseline (Time 1), and 12 (7.17) at follow-up (Time 2), with a statistically significant decrease in reported ISI scores from Time 1 to Time 2 (*t* = −3.04; *p* = 0.003); however, ISI mean scores at both Time 1 and Time 2 were in the Subthreshold Insomnia range (ISI score 8–14), suggesting that this result may not be clinically significant. Data from 84 patients with complete data sets were isolated and compared between baseline (Time 1) and follow-up (Time 2) for additional analyses. Table 5 describes the change in ISI, MOCA, DKEFS Inhibition/Switching, GAD-7, PHQ-8, PSS, PHQ-15, CFQ, and CFT from baseline to Time 2. As noted above, the ISI average score (SD) was 14 (6.48) at Time 1 and 12 (7.17) at Time 2. At baseline (Time 1), the MOCA average score (SD) improved from 25 (3.18) to 26 (2.68) at follow-up (Time 2). The DKEFS Inhibition/Switching baseline average score (SD) was improved from 95 (19.3) to 99 (18.21) at follow-up and trending towards significance. The GAD-7 baseline average score (SD) was improved from 9 (6.12) to 7 (5.94) at follow-up. The PHQ-8 baseline average score (SD) was improved from 13 (5.62) to 11 (6.03) at follow-up. The PSS baseline average score (SD) was improved from 22 (8.28) to 19 (8.76) at follow-up. The PHQ-15 baseline average score (SD) was improved from 13 (5.35) to 12 (5.76) at follow-up. The CFQ baseline average score (SD) was improved from 50 (19.21) to 46 (19.59) at follow-up. The CFS baseline average score (SD) was improved from 64 (18.53) to 74 (24.43) at follow-up. Lower scores on questionnaires, including ISI, GAD-7, PHQ-8, PSS, PHQ-15, and CFQ, indicate improvement, while higher scores on the CFS questionnaire, the MOCA, and the DKEFS Inhibition/Switching task indicate improvement.

Overall, results indicate statistically significant improvements with small effect sizes in insomnia as measured by ISI scores from Time 1 to Time 2 (*t* = −3.04; *p* = 0.003; *d* = 0.33), global cognition as measured by the MOCA scores from Time 1 to Time 2 (*t* = −3.54; *p* < 0.001; *d* = −0.36), and cognitive inhibition and mental flexibility as measured by the DKEFS Inhibition/Switching scores from Time 1 to Time 2 (*t* = −1.94; *p* = 0.055; *d* = −0.18). There were statistically significant improvements with medium effect sizes in fatigue as noted by the CFS scores from Time 1 to Time 2 (*t* = −4.99; *p* < 0.001; *d* = 0.54). Additionally, results indicate statistically significant improvements with small effect sizes in anxiety as measured by the GAD-7 (*t* = 3.93; *p* < 0.001; *d* = 0.40), depression as measured by the PHQ-8 (*t* = 4.20; *p* < 0.001; *d* = 0.41), perceived stress as measured by the PSS (*t* = 4.06; *p* < 0.001; *d* = 0.41), somatic symptoms as measured by the PHQ-15 (*t* = 3.70; *p* < 0.001; *d* = 0.41), and self-reported cognitive failures as measured by the CFQ (*t* = 2.72; *p* = 0.008; *d* = 0.28) scores from baseline (Time 1) to follow-up (Time 2).

## 4. Discussion

Ultimately, the findings of this study indicate that insomnia is a highly prevalent and debilitating symptom of Long COVID. This study was an initial attempt to further describe the presentation of insomnia in a clinical population of Long COVID patients, using a comprehensive, well-validated battery of psychological and neuropsychological assessment measures. Specifically, the current study met its intended aims by: (1) showing a high prevalence (78%) of reported insomnia in Long COVID patients, with nearly 50% of patients endorsing a clinically significant threshold of Moderate Insomnia (ISI score 15–21) or Severe Clinical Insomnia (ISI score 22–28); Long COVID (2) finding that neither infection nor the severity of acute, initial COVID-19 nor the number of COVID-19 infections was correlated with the severity of insomnia or global cognitive function at baseline or follow-up. However, of interest was that global cognitive performance was significantly correlated with ISI at both baseline and follow-up, suggesting that insomnia may be a mediating factor in the cognitive impairment that accompanies Long COVID; Long COVID (3) determines the presence of significant associations between severity of insomnia and reported non-white race, objective and subjective cognitive impairments, and higher levels of anxiety, depression, perceived stress, somatic symptoms, and fatigue. Long COVID and (4) showing mild but statistically significant improvements in insomnia, objective and subjective cognition, anxiety, depression, perceived stress, and somatic symptoms from baseline to 9-month follow-up and moderate improvement in fatigue for Long COVID patients. This suggests that symptoms improve over time in the natural trajectory of Long COVID, but not to a clinically meaningful level. For example, 68% of patients at follow-up continued to endorse insomnia symptoms, with 38% continuing to meet a clinical threshold of insomnia.

Given the aforementioned findings, it is of interest whether acute insomnia related to the initial COVID-19 infection may be the impetus for a cascade of cognitive and emotional issues if left untreated. Further study and use of known efficacious interventions for insomnia in the early stages of COVID-19 infection recovery would provide further clarification of the role of insomnia in Long COVID.

Given the high prevalence of insomnia in patients with Long COVID and the association between insomnia and cognitive issues, mental health issues, and other physiological symptoms of Long COVID, early identification and implementation of interventions to alleviate sleep disruption are of pressing need. At minimum, there is benefit from educational campaigns addressing the connection between Long COVID and insomnia, along with best practices for treating insomnia symptoms. Educational campaigns could target patients with recent COVID-19 infection for prophylactic treatment of persistent insomnia and patients with Long COVID for active management of insomnia symptoms. Interdisciplinary educational materials should also be provided to the myriad primary and specialty care physicians involved in the care of Long COVID patients.

This study also examined the relationship between the severity of acute COVID-19 infection and the severity of insomnia in Long COVID. Interestingly, the severity of acute COVID-19 infection and the number of reported COVID-19 infections were not associated with insomnia severity at either baseline or follow-up for Long COVID patients. Additionally, neither the severity of acute COVID-19 infection nor the number of reported COVID-19 infections was associated with global cognitive performance in Long COVID, as measured by the Montreal Cognitive Assessment (MoCA). However, global cognitive performance was significantly correlated with the severity of insomnia at both baseline and follow-up.

Of interest is whether interventions to alleviate insomnia could also serve to improve “brain fog” and cognitive function in patients with Long COVID symptoms. Long COVID displays similar polysomnography to chronic insomnia in its detrimental impact on sleep efficiency, duration, and waking after sleep onset [20]. Future research could address the utility of behavioral sleep medicine interventions, such as cognitive behavioral therapy for insomnia (CBT-I), and other identified efficacious treatments for chronic insomnia on the improvement in both insomnia and cognitive symptoms in patients with Long COVID. Treatment of insomnia symptoms could ultimately serve to reduce the impact of mental health issues and cognitive issues on quality of life and job performance in Long COVID patients.

Lastly, this study was unique in that it examined the natural trajectory of insomnia in a sample of Long COVID patients at an AMC. At baseline (Time 1), the ISI average score (SD) was 14 (6.48) and 12 (7.17) at follow-up (Time 2). There was a statistically significant decrease in reported ISI scores from Time 1 to Time 2; however, ISI mean scores remained in the Subthreshold Insomnia range (ISI score 8–14) at Time 2. Although the findings of this study suggest statistical improvement in insomnia symptoms over time for Long COVID patients, this improvement may not be clinically meaningful. Insomnia symptoms were still present at time 2, with 68% of patients still reporting some level of insomnia and 12% of patients reporting clinically severe insomnia at follow-up. These findings provide further support for the importance of targeted insomnia interventions for Long COVID patients.

In terms of limitations of the current study, recent investigations suggest that self-report measures and objective measures of sleep quantity and quality do not consistently correlate as expected [34]. Thus, future studies may explore different combinations of insomnia and sleep quality measures, such as actigraphy. This sample was also a clinical sample of convenience, including Long COVID patients seeking care for reported emotional and cognitive symptoms. This sample may differ from other Long COVID populations. Importantly, our sample is composed of primarily white and female participants, a major overrepresentation of these specific groups relative to the demographics of the region in which the study was conducted [35]. This misalignment is not unique to the present study but a reality of academic medical research in general and may limit the generalizability of findings to the larger population [36]. Evidence suggests that racial and ethnic minorities are particularly underrepresented in investigations of sleep issues and treatment outcomes. Some evidence indicates that African American patients are less likely to be treated for insomnia than their Caucasian counterparts [37]. Overrepresentation of Caucasian patients in medical research has a long history with roots in systematic disadvantages such as reduced access to medical care. Reduced access can be due to external factors, such as financial hardship limiting resources or having limited time available to pursue healthcare, but may also be due to intrinsic barriers of low behavioral health literacy and prohibitive cultural factors/stigma [38,39]. This is especially of interest given our finding of a significant association between severity of insomnia and reported non-white race.

The implications of this disparity for the healthcare system are clear, as the care burden for Long COVID is substantial [40], and racial minorities may be more at risk to develop Long COVID. Evidence suggests that racial minorities in the United States are more likely to be hospitalized for COVID-19 infection versus treated in primary care [41]. Future study in the area of Long COVID insomnia could work to resolve these disparities in a number of ways, including purposive sampling for diversity and utilization of participatory and qualitative research methods to lend context to inform quantitative study. At minimum, future studies would be enhanced by including qualitative studies in the literature review, thereby engaging with this arm of research in a way that can influence conceptualization and study design to be more realistic and equitable [42,43]. Furthermore, the inclusion of a control group and other recruitment methods, with larger sample sizes, would allow for more robust statistical analyses and conclusions.

Despite the stated limitations, this study is impactful in that it can serve to inform clinical efforts focused on the prevention, early intervention, and management of insomnia in Long COVID patients. The unique multidomain methodology of this study allowed for a more comprehensive review of insomnia in Long COVID patients. While much is still unknown on the pathophysiological origin of insomnia in Long COVID, experts hypothesize that hypothalamic–pituitary–adrenal axis dysfunction is at the core of this symptomatology [44,45]. The disruption of this axis spurred by persistent immune system dysregulation, neuroinflammation, and mitochondrial dysfunction from COVID-19 infection is the proposed neurobiological underpinning that leads to Long COVID insomnia. Moreover, Cox and Olatunji [46] utilized the 3P model of insomnia to describe sleep disturbance and chronic insomnia triggered by the COVID-19 pandemic. Using the 3P framework, it is hypothesized that pandemic-specific predisposing, precipitating, and perpetuating factors work together to induce acute insomnia onset that then transitions to chronic insomnia [47]. Cox and Olatunji [46] discuss perpetuating factors such as changes in technology usage and light exposure, in addition to Long COVID serving as a perpetuating factor in the onset of chronic insomnia. While many go on to return to baseline sleep patterns after a COVID-19 infection, it is hypothesized that those who endure the perpetuating factor of Long COVID are at higher risk of developing chronic insomnia.

As the scientific community works to further clarify the underlying physiological mechanisms of Long COVID, consideration of sleep-focused interventions to improve insomnia and related Long COVID symptoms can serve to improve cognitive, emotional, and specific physiological symptoms of Long COVID, such as fatigue and somatic symptoms. Previous studies have shown that CBT-I contributes to global improvement in quality of life when administered and adhered to appropriately [48]. This result was reinforced in people with chronic heart failure, a population more similar to Long COVID patients than healthy individuals [49]. In a similar vein, insomnia intervention has been recommended for multiple sclerosis patients experiencing cognitive deficits [50]. Additionally, exercise-focused interventions also demonstrate improved ISI scores [51] and brief educational interventions such as sleep hygiene recommendations, stimulus control, and relaxation training are indicated for insomnia in addition to pharmacological options [6]. Moreover, future studies could incorporate qualitative data measures of Long COVID patients’ real-life experiences with insomnia, providing an opportunity to discover new, crucial questions to explore.

## 5. Conclusions

The current study offers novel and critically important information on insomnia, its concomitant factors, and trajectory in Long COVID using well-validated psychological and neuropsychological assessment measures. The majority of Long COVID patients in this study exhibited symptoms of insomnia (78%), with higher levels of insomnia being associated with higher levels of self-reported anxiety, depression, perceived stress, somatic symptoms, fatigue, and perceived cognitive failures, as well as neuropsychological measures of global cognitive function and mental flexibility. Of those reporting insomnia, 30% reported Subthreshold Insomnia, 30% reported Moderate Insomnia, and 18% reported Severe Clinical Insomnia on the ISI. Moreover, although the findings of this study suggest statistical improvement in insomnia symptoms over time for Long COVID patients, this improvement may not be clinically meaningful. Taken together, these data set the stage for further research involving interventions and clinical practices best suited to alleviate insomnia-related issues in the context of Long COVID. This could include screening for insomnia symptoms for all Long COVID patients using the ISI and subsequent intervention of CBT-I and other evidence-based insomnia interventions. This research also highlights the need for a continued focus on Long COVID in general and its impact on patient neuropsychiatric and cognitive health outcomes.

## Figures and Tables

**Table 1:** Sociodemographic characteristics of patients at Time 1 and Time 2 assessments.

Demographic Characteristic	Time 1 *N* = 172		Time 2 *N* = 89	
	*n*	%	*n*	%
Age				
M (SD)	49 (10.18)		51 (9.99)	
Education				
M (SD)	15 (2.61)		15 (2.49)	
Gender				
Female	121	70	61	69
Male	51	30	28	32
Race				
White/Caucasian	133	78	68	77
Black/African American	36	21	19	22
Other	2	1	1	1
ISI Categories				
No insomnia	33	19	24	27
Subthreshold	51	30	27	30
Moderate	51	30	23	26
Severe	31	18	11	12
No data	6	3	4	5

**Table 2:** Neuropsychological battery by cognitive domain.

Cognitive Domain	Neuropsychological Assessment	Brief Description
*Effort and Validity*	WAIS-IV Embedded Reliable Digit Span	Calculated from WAIS-IV Digit Span Forward and Backward trials.
	CVLT-3 Forced Choice	Total number correct in the forced-choice trial of CVLT-3.
*Cognitive Status*	MoCA	Measures global cognitive function at time of assessment.
*Intelligence/Premorbid*	WTAR	Provides an estimate of overall premorbid IQ
*Functioning*		using a word reading list.
*Attention and*	WAIS-IV Digit Span	The examinee listens to a series of numbers and either repeats or manipulates the numbers in their mind to
*Concentration*		correctly respond.
*Processing Speed*	OSDMT	The examinee verbally names the number that matches each unique symbol as quickly as they can.
	OTMT Part A	The examinee counts from 1 to 25 as quickly as they can.
	D-KEFS Color Naming and Word Reading	The examinee names colors and reads words as quickly as they can.
	COWAT FAS	The examinee lists as many words as they can that begin with a single letter in 60 s.
*Executive Function*	OTMT Part B	The examinee switches between counting and listing the letters of the alphabet in order as quickly as they can.
	D-KEFS Inhibition and Inhibition/Switching	The examinee inhibits reading a word to name the color the word is printed in or switches between reading the word and naming the color it is printed in.
*Language*	COWAT Animals	The examinee lists as many animals as they can in 60 s for a measure of semantic verbal fluency.
*Visuospatial Construction*	RBANS Figure Copy	The examinee copies a complex figure as accurately as they can.
*Semantic Memory*	CVLT-3 Standard Form	The examinee listens to a word list and repeats back as many words as they can from the list after 5 learning trials, a brief delay, and a long delay.
*Episodic Memory*	RBANS Story Memory Immediate and Delayed	The examinee listens to a short story and repeats the story back immediately and after a delay.
*Visuospatial Memory*	RBANS Figure Recall	The examinee recalls the previously drawn complex figure from the RBANS Figure Copy subtest.

*Note*: Abbreviations: WAIS-IV, Wechsler Adult Intelligence Scale—Fourth Edition; CVLT-3, California Verbal Learning Test—Third Edition; MoCA, Montreal Cognitive Assessment; WTAR, Wechsler Test of Adult Reading; OSDMT, Oral Symbol Digit Modalities Test; OTMT, Oral Trail Making Test; D-KEFS, Delis–Kaplan Executive Functioning System; COWAT, Controlled Oral Word Association Test; RBANS, Repeatable Battery for the Assessment of Neuropsychological Status.

**Table 3:** Frequency of infection severity.

Infection Severity	*N* = 164	%
*Asymptomatic*	2	1
*Mild*	48	28
*Moderate*	64	37
*Severe*	35	20
*Critical*	15	8

**Table 4:** Correlations between greater reported insomnia symptoms and neurocognitive and self-reported mood/behavior measures.

	1.	2.	3.	4.	5.	6.	7.	8.	9.	10.	11.	12.	13.
1. Gender	--												
2. Age	−0.07	--											
3. Race	0.08	−0.10	--										
4. Education	−0.03	0.02	−0.11	--									
5. MoCA	0.2	−0.06	−0.14	0.37 **	-								
6. DKEFS Inhibition/Switching	−0.04	0.12	−0.17 *	0.29 **	0.32 **	-							
7. GAD-7	0.08	−0.23 **	0.15 *	−0.13	−0.25 **	−0.26 **	-						
8. PHQ-8	0.13	−0.18 *	0.11	−0.11	−0.22 **	−0.29 **	0.71 **	-					
9. PSS	0.17 *	−0.24 **	0.14	−0.12	−0.19 *	−0.28 **	0.75 **	0.73 **	-				
10. PHQ-15	0.22 **	−0.16 *	0.03	−0.12	−0.21 **	−0.19 *	0.43 **	0.54 **	0.47 **	-			
11. ISI	0.03	−0.14	0.24 **	−0.07	−0.21 **	−0.17 *	0.41 **	0.52 **	0.38 **	0.51 **	-		
12. CFQ	0.23 **	−0.19 *	0.13	−0.26 **	−0.23 **	−0.28 **	0.54 **	0.61 **	0.69 **	0.46 **	0.41 **	-	
13. CFS	−0.18 *	0.06	−0.001	0.10	0.09	0.21 **	−0.44 **	−0.64 **	−0.48 **	−0.59 **	−0.46 **	−0.55 **	-

*Note:* * *p* < 0.05, ** *p* < 0.01. *N* = 172. Only demographic factors and variables with significant relationships are provided. The following measures were not significant: OSDMT, WAIS-IV Digit Span, OTMT, D-KEFS Color Naming, D-KEFS Word Reading, D-KEFS Inhibition, CVLT-3 Brief Form, RBANS Story Memory, and Story Memory Delayed Recall.

**Table 5:** ISI differences in values from baseline and follow-up visits.

Score	N	Mean (SD)	t	*p*-Value
ISI				
Baseline	84	14 (6.48)		
Time 2	84	12 (7.17)	3.04	0.003 **
MOCA				
Baseline	90	25 (3.18)		
Time 2	90	26 (2.68)	−3.54	<0.001 **
DKEFS Inhibition/Switching				
Baseline	87	95 (19.3)		
Time 2	87	99 (18.21)	−1.94	0.055
GAD-7				
Baseline	86	9 (6.12)		
Time 2	86	7 (5.94)	3.93	<0.001 **
PHQ-8				
Baseline	86	13 (5.62)		
Time 2	86	11 (6.03)	4.20	<0.001 **
PSS				
Baseline	85	22 (8.28)		
Time 2	85	19 (8.76)	4.06	<0.001 **
PHQ-15				
Baseline	84	13 (5.35)		
Time 2	84	12 (5.76)	3.70	<0.001 **
CFQ				
Base line	84	50 (19.21)		
Time 2	84	46 (19.59)	2.72	0.008 **
CFS				
Baseline	85	64 (18.53)		
Time 2	85	74 (24.43)	−4.99	<0.001 **

*Note:* SD, standard deviation. ** *p* < 0.01. Scores for insomnia include 0–7 (no clinically significant insomnia), 8–14 (subthreshold insomnia), 15–21 (clinical insomnia, moderate), and 22–28 (clinical insomnia, severe).

## Data Availability

The original contributions presented in this study are included in the article. Further inquiries can be directed to the corresponding author.
